# GC-MS Metabolomics to Evaluate the Composition of Plant Cuticular Waxes for Four *Triticum aestivum* Cultivars

**DOI:** 10.3390/ijms19020249

**Published:** 2018-01-23

**Authors:** Florent D. Lavergne, Corey D. Broeckling, Darren M. Cockrell, Scott D. Haley, Frank B. Peairs, Courtney E. Jahn, Adam L. Heuberger

**Affiliations:** 1 Department of Horticulture and Landscape Architecture, Colorado State University, Fort Collins, CO 80523, USA; florent.lavergne@colostate.edu; 2 Proteomics and Metabolomics Facility, Colorado State University, Fort Collins, CO 80523, USA; corey.broeckling@colostate.edu; 3 Department of Bioagricultural Sciences and Pest Management, Colorado State University, Fort Collins, CO 80523, USA; darren.cockrell@colostate.edu (D.M.C.); frank.peairs@colostate.edu (F.B.P.); 4 Department of Soil and Crop Sciences, Colorado State University, Fort Collins, CO 80523, USA; scott.haley@colostate.edu

**Keywords:** gas chromatography-mass spectrometry, scanning electron microscopy, cuticular wax, epicuticular wax crystal

## Abstract

Wheat (*Triticum aestivum* L.) is an important food crop, and biotic and abiotic stresses significantly impact grain yield. Wheat leaf and stem surface waxes are associated with traits of biological importance, including stress resistance. Past studies have characterized the composition of wheat cuticular waxes, however protocols can be relatively low-throughput and narrow in the range of metabolites detected. Here, gas chromatography-mass spectrometry (GC-MS) metabolomics methods were utilized to provide a comprehensive characterization of the chemical composition of cuticular waxes in wheat leaves and stems. Further, waxes from four wheat cultivars were assayed to evaluate the potential for GC-MS metabolomics to describe wax composition attributed to differences in wheat genotype. A total of 263 putative compounds were detected and included 58 wax compounds that can be classified (e.g., alkanes and fatty acids). Many of the detected wax metabolites have known associations to important biological functions. Principal component analysis and ANOVA were used to evaluate metabolite distribution, which was attributed to both tissue type (leaf, stem) and cultivar differences. Leaves contained more primary alcohols than stems such as 6-methylheptacosan-1-ol and octacosan-1-ol. The metabolite data were validated using scanning electron microscopy of epicuticular wax crystals which detected wax tubules and platelets. Conan was the only cultivar to display alcohol-associated platelet-shaped crystals on its abaxial leaf surface. Taken together, application of GC-MS metabolomics enabled the characterization of cuticular wax content in wheat tissues and provided relative quantitative comparisons among sample types, thus contributing to the understanding of wax composition associated with important phenotypic traits in a major crop.

## 1. Introduction

Common wheat (*Triticum aestivum* L.) is a widely grown staple crop, and is often affected by biotic and abiotic pressure that reduces grain yield [1]. In wheat, resistance to stress is associated with metabolic responses in various tissues and organs, changing the plant chemical composition of tissues that provide stress tolerance. The surface of leaf and stem organs is known as the cuticle, which is a primary point of contact with insects and pathogens, and regulates water retention during periods of high temperature, low atmospheric humidity, and low soil moisture [2,3,4]. The cuticle is an extracellular matrix of two main components: cutin (a simple polymer) and lipids, termed epicuticular waxes (herein referred to as “waxes”) [5]. Waxes are considered end-products of long-chain lipid metabolism and include alkanes, fatty acids, primary and secondary alcohols, ketones, esters, and aldehydes [6].

Waxes are important to stress tolerance for their role in forming a physical barrier to prevent dust and debris accumulation on hydrophilic surfaces and form a natural obstacle to pathogen penetration [5,7]. Cuticular fatty acids and primary alcohols have been demonstrated to facilitate resistance of cabbage (*Brassica oleracea*) to diamondback moth larvae (*Plutella xylostella*), specifically through chemical deterrence [8]. Waxes can also act as photoprotectants for ultraviolet light [9]. Likewise, wax composition (e.g., proportions of ketones and primary alcohols) and content correlate with glaucousness, a bluish-green appearance of the plant cuticle that is associated with drought tolerance [10,11,12,13,14].

Waxes assemble in the cuticle as three-dimensional crystals of 23 types based on micromorphology [15]. Scanning electron microscopy (SEM) studies reveal that the “platelet” and “tubule” types of epicuticular wax crystals are distributed among all major groups of plants [12,15]. Further, the wax composition (lipid class and abundance) is associated with the type of wax crystal. In *Triticum* species, platelets are mostly composed of primary alcohols [12], whereas tubules are high in content of β-diketones [15,16]. Here, these associations are used to validate the accuracy of a customized biochemical analysis workflow on wax detection.

Various methods have been developed to evaluate waxes. Leaf or stem samples are usually air- or freeze-dried and then immersed in a polar (e.g., methanol) or non-polar (e.g., hexane) solvent [17,18,19,20]. Rapid dips in hexane can solubilize wax alkanes, primary alcohols, fatty acids, ketones, and aldehydes that are often known for their agronomic importance [10,21,22,23]. Further, several studies have compared gas chromatography injection methods (e.g., split ratios) and oven temperatures necessary for wax compounds [24,25,26].

In plants, metabolomics is the comprehensive analysis of small molecules (~50–1200 Da), typically involved in primary or specialized (secondary) metabolism. While waxes are in fact end-products of metabolism (they are exuded from leaf and stem cells), they are amenable to analysis by metabolomics methods due to their chemical properties. A common method to evaluate primary metabolites in plants is to extract, derivatize via silylation, and detect metabolites using gas chromatography coupled to mass spectrometry (GC-MS) [27]. Many plant waxes are small and semi-volatile, and are also detected using GC-MS [11,13,24,28,29,30]. Further, many contain hydroxyl groups that allow for derivatization to improve volatility and detection by GC-MS.

In MS-based non-targeted metabolomics, extracts from different samples are analyzed as metabolite “profiles”, determined by a collection of molecular features that include chromatographic retention indices (RI, derived from retention times), mass-to-charge ratios (*m*/*z*) and the relative abundance of each metabolite [31,32]. Non-targeted metabolomics methods utilize data processing algorithms that attempt to align large datasets (chromatographic retention times can drift over time) and provide information on all detectable *m*/*z*. Due to the complexity of calculating absolute quantities for thousands of compounds, metabolite quantities are recorded as relative abundances [33,34], typically to the total chemical signal or an internal standard. The resulting data matrix is unique in that it allows for a comparison of chemical profiles to evaluate sources of variation in a biological system.

In this study, we combine the amenability of GC-MS for waxes with non-targeted metabolomics data processing methods to enable comparisons of wax composition on tissues (i.e., leaf and stem cuticles) of four wheat cultivars that differ for glaucousness and resistance to an insect pest. Further, micro-morphological features are evaluated using SEM to validate the relevance of our workflow. Here, we provide a comprehensive characterization of wheat waxes and demonstrate variation among tissues and cultivars. The data supports the potential to breed for wax traits with demonstrated effects on a variety of biologically relevant phenotypes, including stress resistance. Further, this method allows for high-throughput extraction, detection, and quantitation of waxes and is applicable to very large sample sets made up of thousands of metabolites.

## 2. Results

### 2.1. Cuticular Waxes Detected on Leaf and Stem Cuticles of Wheat

**Figure 1:**
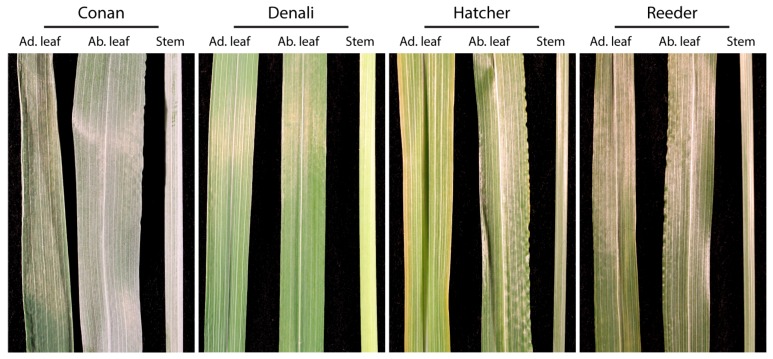
Glaucousness degree on leaf and stem cuticles of four wheat cultivars. Photographs showing a bluish-green appearance (high glaucousness) for the cultivar Conan on both sides of its leaves, and stems. Denali, Hatcher, and Reeder cuticular tissue showed lower levels of glaucousness. All pictures were taken from plants at Zadoks stage ca. 55. Abbreviations: ad. leaf = adaxial leaf surface; ab. leaf = abaxial leaf surface.

We focused on four cultivars for their phenotypic variation in: (1) resistance to the wheat stem sawfly (*Cephus cinctus* Norton, WSS) [30,35]; (2) degree of surface glaucousness (Figure 1); and (3) growth habit (spring and winter wheat types).

A total of 263 putative compounds were detected using GC-MS metabolomics. Sixty-nine of the 263 putative compounds were annotated as plant metabolites using retention index RI (derived from retention time as described in the materials and methods section) and mass spectral matching, including 58 cuticular waxes (Table 1). Compounds were sorted and numbered in Table 1 based on their chemical class and RI. Bolded numbers in the results and discussion sections correspond to metabolites in Table 1 and Table 2. Additional information regarding compound annotation can be found in the supplementary materials (Table S1). Twenty cuticular wax metabolites were identified as alkanes, ranging from C_20_ (eicosane, **1**, RI = 2000) to C_42_ (dotetracontane, **20**, RI = 4200). Eleven were straight-chain alkanes and nine were methyl-branched at various positions. Fifteen alkanes varied in content between leaf and stem cuticles and 11 varied among the four cultivars (ANOVA, FDR adjusted *p* < 0.05). Twenty-two fatty acids were detected and ranged from C_7_ (heptanoic acid, **21**, RI = 1044) to C_34_ (tetratriacontanoic acid, **42**, RI = 3037). Nineteen of the fatty acids were straight chain, including three compounds with one or more double bonds and three fatty acids that were methyl-branched. Fourteen fatty acids varied between tissues and only tetratriacontanoic acid had significant variation in content among cultivars (ANOVA, FDR adjusted *p* < 0.05). Six ketones were detected and ranged from C_9_ (nonane-4,6-dione, **43**, RI = 1124) to C_35_ (pentatriacontan-2-one, **48**, RI = 2881), and included two monoketones and four β-diketones. Five ketones significantly varied between leaf and stem cuticles, and tritriacontan-3-one was the only monoketonic structure to vary in content among cultivars (ANOVA, FDR adjusted *p* < 0.05). Ten wax compounds were annotated as primary alcohols and ranged from C_22_ (docosan-1-ol, **49**, RI = 2044) to C_33_ (tritriacontan-1-ol, **58**, RI = 2855). Eight primary alcohols were straight chain metabolites and two were methyl-branched. Out of the ten alcohols, eight showed a statistically significant difference in content between tissues and six differed among cultivars (ANOVA, FDR adjusted *p* < 0.05).

The remaining 11 cuticular compounds out of the 69 annotated metabolites detected in the extract did not represent typical waxes (Table 2). These included three carboxylic acids (**59**–**61**), two carbohydrates (**62**,**63**), one primary amine (**64**), two polycyclic ketones (**65**,**66**), one ester (**67**), one flavonoid (**68**), and one sterol (**69**). Their carbon structure ranged from C_4_ to C_29_ and were characterized by retention indices of 1192 to 2885. Between tissues, six of the non-wax metabolites varied in content and two showed differences among cultivars.

Nine of the 58 annotated wax metabolites were identified in the literature as being involved in plant–insect interactions, and included alkanes, fatty acids, and primary alcohols (Table 1). Further evidence of the biological importance of waxes included antibiotic activity for tetradecanoic acid (**22**) and allelopathic activity for hexacosanoic acid (**33**) [37,43]. Tetracosanoic acid (**32**) has been described as a major wax precursor and hentriacontane-14,16-dione (**46**) is associated with a high degree of glaucousness in wheat [42,45]. Another application of the variation in wax composition is the classification of plants based on biochemical differences (i.e., chemotaxonomy). A total of seven alkanes (e.g., tricosane, **2**; pentacosane, **5**) detected in our study were previously shown to be functional chemotaxonomy markers [24].

### 2.2. Cuticular Wax Composition in Leaves and Stems of Wheat

**Figure 2:**
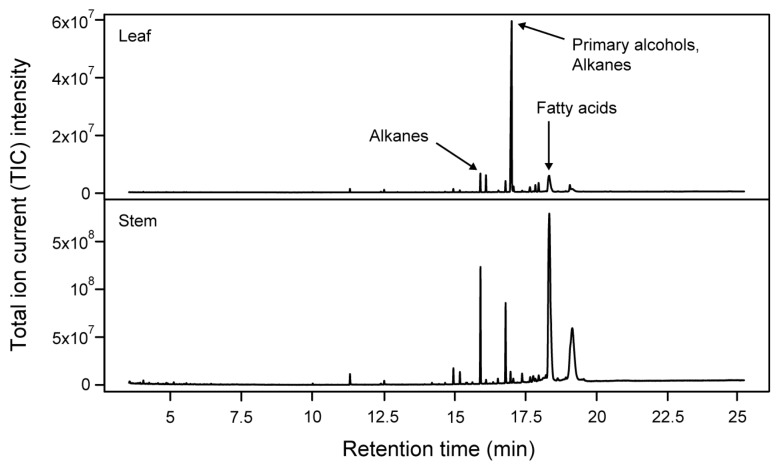
Example GC-MS chromatograms for leaf and stem metabolites in the wheat cultivar Hatcher. Arrows indicate which class of compounds was the most represented at the respective retention times.

**Figure 3:**
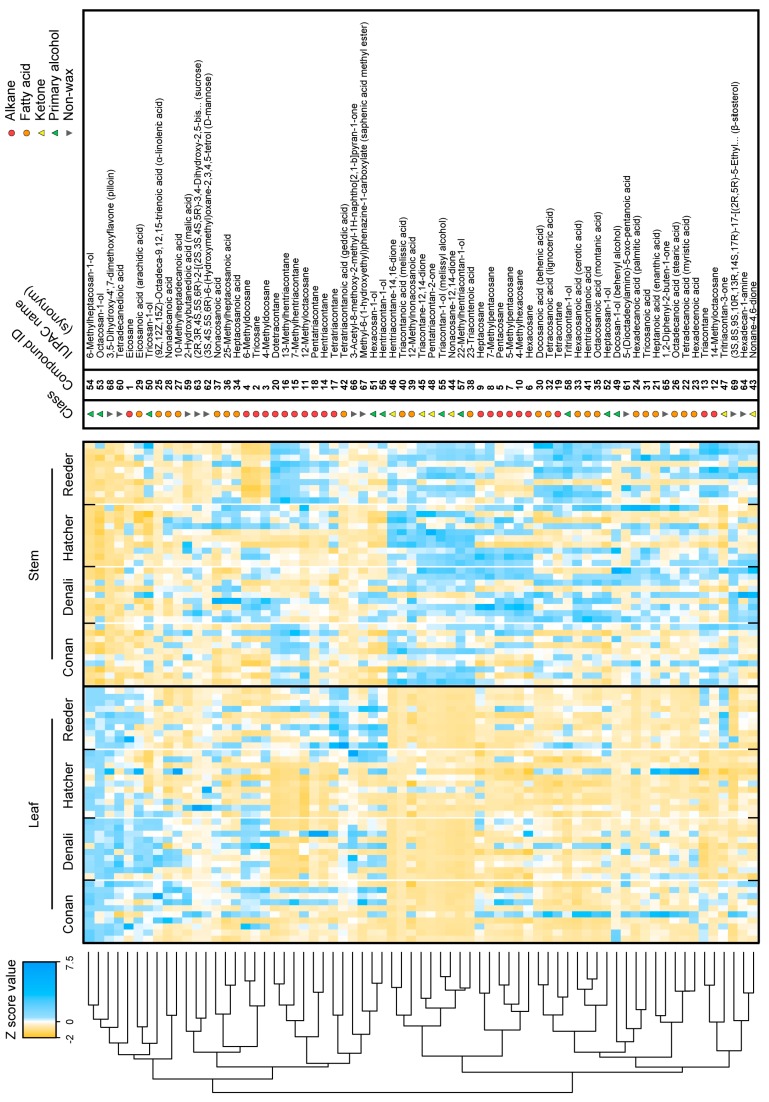
Metabolite levels in wheat. Heat map showing metabolite levels on leaf and stem cuticles of wheat. Composition often was independent of cultivar, and two main clusters were identified: metabolites higher in content on leaf cuticles and lower on stem cuticles (**top**), and metabolites lower in content on leaf cuticles and higher on stem cuticles (**bottom**). The 58 wax and 11 non-wax metabolite contents were z-transformed, subjected to hierarchical clustering, and displayed as color (blue = high content, yellow = low content). Each cell represents the z transformed content of a single biological replicate for a total of *n* = 9–11 replicates/cells per cultivar. Z-transformation was based on the mean abundance and standard deviation of the metabolite across all samples.

**Figure 4:**
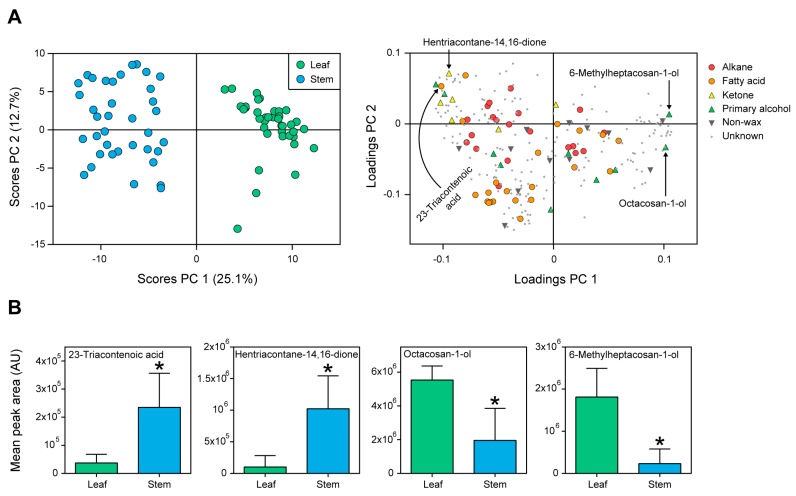
Metabolite distribution in leaf and stem cuticles. Multivariate analysis demonstrating specific composition in cuticular wax chemistry between leaf and stem surfaces of wheat. (**A**) Principal component analysis (PCA) of the four cultivars showed that most metabolite (wax and non-wax) variation was due to differences between leaves and stems (principal component, or PC scores, left). Each PC score point represents the metabolite profile for a single biological replicate (*n* = 9–11 replicates per cultivar). Loadings were colored by wax type and include primary alcohols, ketones, and fatty acids. Example wax metabolites are indicated by arrows. (**B**) Box plots of example waxes that varied between leaf and stem cuticles. Metabolite values are reported as the mean content across all cultivars ± standard error of the mean (*n* = 40 biological replicates per tissue). Asterisks indicate variation between tissues (ANOVA, FDR-adjusted *p* < 0.05). Abbreviations/Notations: PC = principal component; AU = arbitrary unit; unknown = unknown metabolite, no annotation.

The general appearance of metabolite profiles between leaf and stem samples of wheat was displayed using example chromatograms in Figure 2. Chromatograms illustrate the distribution in time (retention time) and the total ion current (TIC) intensity of all the putative compounds. Further, the content of the 69 annotated metabolites (including the 58 cuticular waxes) was z-transformed and displayed using a heat map combined with hierarchical clustering (Figure 3). The heat map highlights variation in content for most of the metabolites between tissues. Principal component analysis (PCA) also demonstrated significant variation between tissues (Figure 4A, left; Principal component **1**, or PC 1, 25.1% of the variation) due to fatty acids and β-diketones (Figure 4A, right; PC 1 loadings).

As an example, four wax metabolites that contributed to the PCA model were labeled (Figure 4A, right), discussed as they appear from left to right and displayed as box plots to demonstrate variation between tissues (Figure 4B). Fatty acid 23-triacontenoic acid (**38**, FDR adjusted *p* = 1.39 × 10^−14^) and β-diketone hentriacontane-14,16-dione (**46**, FDR adjusted *p* = 1.29 × 10^−15^) were higher on stems and contributed to the tissue separation for PC 1 (loading scores plot). The primary alcohols octacosan-1-ol (**53**, Student’s *t*-test, FDR adjusted *p* = 3.91 × 10^−16^) and 6-methylheptacosan-1-ol (**54**, Student’s *t*-test, FDR adjusted *p* = 3.28 × 10^−19^) were higher in content on leaf than stem surfaces and highly contributed to the separation of tissues for PC 1 as well.

### 2.3. Wheat Cuticular Wax Composition among Cultivars

A further analysis of the heat map (Figure 3) demonstrates metabolite variation of the four wheat cultivars Conan, Hatcher, Reeder, and Denali. As an example, several alkanes were shown to be lower in content on Reeder stem cuticles compared to other cultivars (tricosane, **2**, FDR adjusted *p* = 1.9 × 10^−4^; 4-methyldocosane, **3**, FDR adjusted *p* = 2.6 × 10^−11^; 6-methyldocosane, **4**, FDR adjusted *p* = 9.15 × 10^−6^). Likewise, Hatcher and Reeder leaf surfaces contained less (9*Z*,12*Z*,15*Z*)-octadeca-9,12,15-trienoic acid (**25**, FDR adjusted *p* = 5.25 × 10^−3^), 10-methylheptadecanoic acid (**27**, FDR adjusted *p* = 1.6 × 10^−10^), and nonadecanoic acid (**28**, FDR adjusted *p* = 5.49 × 10^−4^) compared to Conan and Denali.

**Figure 5:**
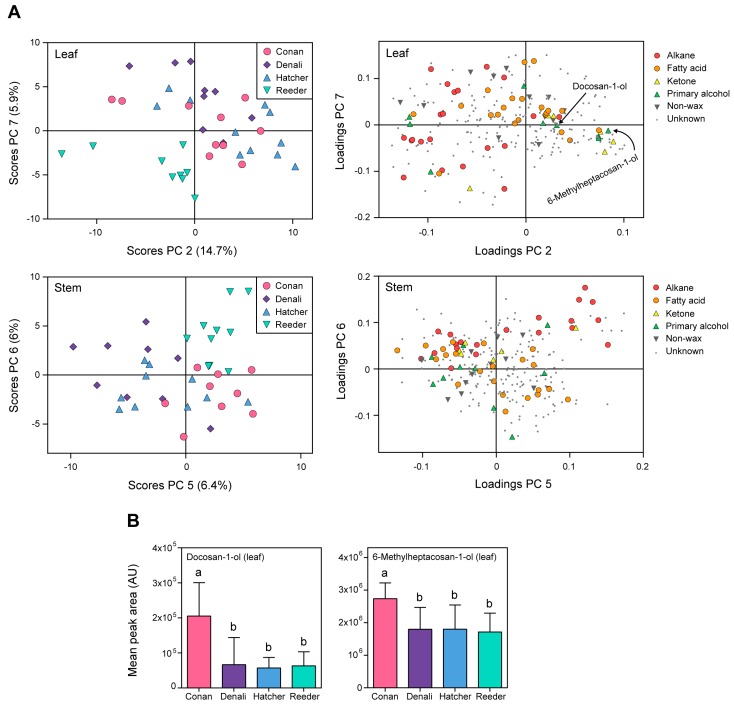
Metabolite levels among cultivars within leaf and stem cuticles. Multivariate analysis showing cuticular wax variation among different cultivars of wheat. (**A**) Principal component analysis showed cultivar variation within leaf (**top**) and stem (**bottom**) surfaces. Loadings indicate metabolites involved in the separation of cultivars and are colored based on wax type. Metabolites denoted on the PCA loadings plot exhibited increased content in the cultivar Conan. (**B**) Box plots of two wax metabolites that were higher in content on Conan leaf cuticles. Metabolite values are reported as the mean content of leaf cuticles for each cultivar ± standard error of the mean (*n* = 9–11 replicates per cultivar). Lowercase letters indicate variation among cultivars (ANOVA, Tukey HSD post-hoc FDR-adjusted *p* < 0.05). Abbreviations/Notations: PC = principal component; AU = arbitrary unit; unknown = unknown metabolite, no annotation.

The significant variation in wheat cuticular compounds including waxes was further supported by PCA that showed separation among cultivars on leaf and stem cuticles (Figure 5). A total of 11 PCs were generated in the PCA model explaining 77% of the variation. For leaves, the largest separation among cultivars appeared for the combination of PC 2 (that explained 14.7% of the total variation in the sample set) and PC 7 (5.9% of the variation) (Figure 5A, upper left). Twenty (34%) of the annotated waxes were associated with the separation of cultivars within leaves (for all 20 metabolites, ANOVA, FDR-adjusted *p* < 0.05). For stems, the largest separation among cultivars was observed for the combination of PC 5 (6.4% of the total variation) and PC 6 (6% of the variation) (Figure 5A, bottom left). Twenty-three of the annotated cuticular waxes (40%) were associated with the separation of cultivars for metabolites within stems (for all 23 wax metabolites, ANOVA, FDR-adjusted *p* < 0.05).

Conan, the glaucous cultivar (Figure 1) with resistance to the wheat stem sawfly [30,35], had no wax metabolites that were unique on its leaf or stem cuticles (i.e., presence/absence). However, several individual metabolites were greater in abundance compared to the other three cultivars, such as the primary alcohols docosan-1-ol (**49**) and 6-methylheptacosan-1-ol (**54**) (Figure 5A,B, compounds denoted in the PC loadings plot for leaves, from left to right). An ANOVA followed by Tukey HSD pairwise comparisons revealed variation for docosan-1-ol (**49**) (FDR adjusted *p* = 2.22 × 10^−3^) and 6-methylheptacosan-1-ol (**54**) (FDR adjusted *p* = 1.29 × 10^−2^) on Conan leaf surface (Figure 5B).

### 2.4. Association of Epicuticular Wax Content and Crystal Microstructure in Wheat

**Figure 6:**
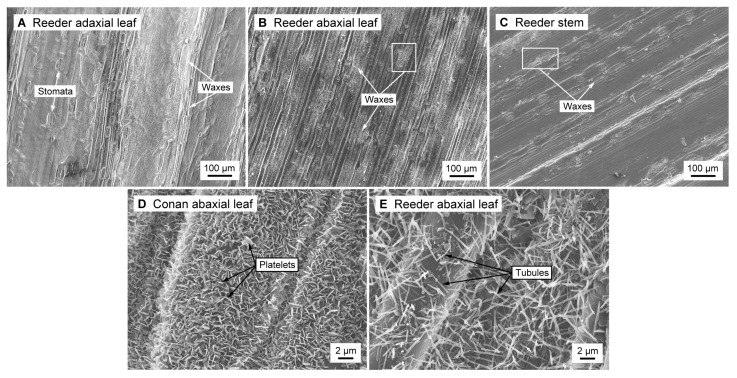
Epicuticular wax crystal variation on the surface of greenhouse-grown wheat. Scanning electron micrographs of wheat epicuticular wax crystals showed that: (**A**) Reeder adaxial leaf surface had the highest content in wax crystals; (**B**) Reeder abaxial leaf surface displayed intermediate wax crystal content; (**C**) Reeder stem had low wax crystal content; (**D**) Conan abaxial leaf surface was exclusively made of platelet crystals; and (**E**) Reeder abaxial leaf surface consisted of tubules.

Scanning electron microscopy (SEM) was performed to characterize wax microstructural variation. This supplementary procedure aimed at validating the consistency of the metabolomics data. Leaf and stem cuticle tissue from mature plants was collected, air-dried, and micrographs of the adaxial leaf surface (upper side), abaxial leaf surface (lower side), and stem were obtained for the four cultivars (Figure 6). It is important to note that our study was limited to growing spring wheat cultivars (Conan and Reeder) in a greenhouse and winter wheat cultivars (Denali and Hatcher) were sampled from the field. While the growing location (i.e., controlled versus non-controlled environments) may contribute to variation in wax content and crystal microstructure, no clear trends were observed between the field- and greenhouse-grown plants.

Based on semi-quantitation of the epicuticular wax content (measure of percent wax abundance on the cuticle total surface using image processing software, Figure S1), microscopy observations revealed specific composition among adaxial and abaxial leaf surfaces and stems. As an example, Reeder adaxial leaf micrographs displayed more epicuticular wax crystals than Reeder abaxial leaf or stem micrographs (Figure 6A–C). The adaxial leaf surface of all cultivars was exclusively covered by platelets, whereas stems had only tubules. However, variation among cultivars was observed for the abaxial leaf surface whereby Conan had only platelets (Figure 6D) whereas Reeder (Figure 6E), Hatcher, and Denali had tubules.

## 3. Discussion

### 3.1. Many of the Detected Cuticular Waxes Have Specialized Biological Functions

The work presented herein characterized the chemical composition of cuticular waxes of *Triticum aestivum* on leaf and stem cuticles and among different cultivars. Many of the detected waxes have specialized functions related to important phenotypic traits. Antibiotic (tetradecanoic acid, **22**) and allelopathic (hexacosanoic acid, **33**) activities have been described for plant waxes [37,43]. Seven alkanes detected in our study, including tricosane (**2**) and pentacosane (**5**), are known as functional chemotaxonomy markers [24]. Further, alkane, fatty acid, and primary alcohol waxes (e.g., eicosane, **1**; hexadecanoic acid; **24**, octacosan-1-ol, **53**) have been shown to stimulate or interfere with insect attachment and oviposition, including the wheat/hessian fly interaction [10], and the seven-spotted ladybug (*Coccinella septempunctata*) interaction with bioinspired wax surfaces [36]. Moreover, in common wheat*,* high β-diketone (e.g., hentriacontane-14,16-dione, **46**) content was associated with glaucousness and drought resistance [3,45]. In [39], hentriacontane-14,16-dione (**46**) was absent in waxless, English grain aphid (*Sitobion avenae*) susceptible Triticale (×*Triticosecale*) cultivars, and present in high content in waxy and resistant Triticale cultivars, supporting its role in plant–insect interaction.

The heat map (Figure 3), PCA and univariate analyses (Figure 4 and Figure 5) revealed higher contents of two primary alcohols on leaves of Conan, namely docosan-1-ol (**49**) and 6-methylheptacosan-1-ol (**54**), compared to Reeder, Denali, and Hatcher. Further, increased platelet content on the abaxial leaf surface (Figure 6D) was observed in this cultivar. The glaucous appearance of a plant tissue is mostly due to β-diketones and primary alcohols, and wax matrices with increased primary alcohols can result in the formation of dense crystal platelets that contribute to drought stress resistance [3,12,46]. The genetic regulation of hexaploid wheat wax biosynthesis has been partially elucidated, and studies have identified quantitative trait loci that contribute to wax phenotypes. Specifically, wax synthesis and glaucousness loci (*W1* and *W2*), along with loci coding for their inhibitors (*Iw1* and *Iw2*), were genetically mapped using wheat genomic DNA from leaf cells [47]. Another locus of interest is the one that contains the *W3* gene [45]. *W3* facilitates biosynthesis of β-diketones, where the *Iw1* gene codes for an inhibitor of β-diketone synthesis, and regulation of expression of these genes can influence glaucousness and cuticle permeability [14,17,45]. Further, the *TaFAR* gene family (including *TaFAR1* to *TaFAR4*) regulates the accumulation of primary alcohols [48]. Recently, a study of transcription factors involved in the regulation of wax synthesis genes (*TaWXPL1D* and *TaWXPL2B*) demonstrated differential expression in leaves of two wheat cultivars that contrasted in drought resistance and glaucousness, consequently to water deprivation [49]. The allelic diversity in these genes, and their influence on wax composition and glaucousness, is largely unknown and warrants future investigation that will help elucidate the molecular mechanisms underlying resistance to water stress.

### 3.2. Cuticular Waxes Differed between Leaves and Stems

The heat map (Figure 3) and PCA (Figure 4) showed variation for most individual cuticular wax metabolites. The two waxes that were higher in content on stems were the fatty acid 23-triacontenoic acid (**38**) and β-diketone hentriaconane-14,16-dione (**46**) (Figure 4B). To our knowledge, fatty acid **38** has not been previously reported as a cuticular wax. Compound **46** is known to be associated with glaucousness in common wheat and pest resistance in Triticale [39,45]. Tubule-shaped crystals were encountered on stems along with high content in β-diketones for all cultivars. The high content in β-diketones results in the formation of tubule-shaped crystals that contribute to both the degree of glaucousness and frost resistance [50,51,52].

Leaf cuticles were enriched for primary alcohols compared to stems (Figure 4B). High content of typical platelet-shaped crystals involved in both biotic and abiotic stress resistance (e.g., insect and drought resistance) is due to a primary alcohol-rich content on the plant tissue surface [10,12,46,53]. In this study, higher levels in octacosan-1-ol (**53**) and 6-methylheptacosan-1-ol (**54**) were found on leaf cuticles (Figure 4B). Primary alcohol **53** is a potential insect repellent for the hessian fly and is thus of importance in plant–insect interactions [10], and **54** has not been described in the literature. Further, while the role of high fatty acid content on the stem surface of our wheat cultivars remains to be elucidated, the high content of primary alcohols on leaves and β-diketones on stems supports a potential role in protection of these tissues against biotic and abiotic pressure.

### 3.3. Cuticular Waxes Varied in Composition among the Four Cultivars

The contribution of genetic diversity to variation in cuticular wax composition was investigated by comparing four different cultivars, including two spring and two winter wheat types (Figure 3). The analysis revealed a relatively low content of alkanes tricosane (**2**), 4-methyldocosane (**3**) and 6-methyldocosane (**4**) on Reeder stem surface. Reeder is a spring wheat cultivar widely grown in the Northern Great Plains and broadly adapted for rainfed (non-irrigated) production conditions. Drought stress has been shown to increase levels of alkanes on the leaf surface of alfalfa [54]. Thus, it is possible that Reeder modifies its wax composition upon exposure to drought stress, and potentially increases alkane content for its protective effects.

Multivariate analysis allowed for partial cultivar discrimination based on wax metabolite content (Figure 5A). Wax composition has been shown to vary among plant species, and among cultivars within a species. A study showed that foliage wax of onion (*Allium cepa*) varied among four cultivars, resulting in different degrees of resistance to the onion thrips (*Thrips tabaci*) [55]. The growing region of tussock grass species has been shown to correlate with wax content [56]. The two Australian wheat cultivars RAC875 and Kukri display unique wax compositions that result in different types of epicuticular wax crystals on their abaxial leaf surface [3]. While the abaxial leaf surface of RAC875 was made of tubules, the equivalent tissue in Kukri was exclusively made of platelets. Further, changes at higher taxonomy levels are also well documented, as is the case for two rocktrumpet species (genus *Mandevilla*) that differ in their wax profiles [19]. Together, these studies support that genetic diversity in wax composition may be common among individual cultivars within a plant species.

Conan leaf cuticles displayed high levels of the primary alcohols 6-methylheptacosan-1-ol (**54**) and docosan-1-ol (**49**) (Figure 5B). Although these cuticular waxes have not been described as involved in defense processes, plant leaves that exhibit high content in primary alcohols tend to be covered by epicuticular wax crystals (i.e., platelets) involved in resistance [12,46,53]. The increased production of these two alcohol waxes on Conan leaf cuticles is potentially responsible for reshaping the surface morphology and influencing interactions with the environment and biotic sources.

### 3.4. Cuticular Wax Composition Was Associated with Epicuticular Wax Crystal Microstructure

As previously mentioned, wax chemical composition is associated with wax crystal formation [3]. SEM analysis revealed the presence of tubule and platelet crystals on the cuticle, and variation among tissues and cultivars was observed (Figure 6). Platelet-shaped structures have been observed in major plant groups including various angiosperms and gymnosperms, where tubules made of a significant proportion of β-diketones, as observed in our wax chemical analysis, are commonly found among the Poaceae [12,15].

The wax content of Conan leaf surface was characterized by a higher proportion in primary alcohols docosan-1-ol (**49**) and 6-methylheptacosan-1-ol (**54**). Waxes made of abundant primary alcohols form platelet-shaped crystals on the plant surface [12]. In addition, the primary alcohol octacosan-1-ol (**53**) is associated with plant–insect interactions and leaf glaucousness [10]. Wax composition can influence insect attachment to the plant surface [57], and a study on the carnivorous pitcher plant (*Nepenthes alata*) revealed that a slippery zone on leaves is made of platelet-shaped crystals that detach after the insect touches the tissue, reducing the time of interaction between plant and pest [53]. The cultivar Conan shows a high degree of glaucousness and resistance to the wheat stem sawfly, and our study supports that this may be due to increased wax content, increased content of docosan-1-ol (**49**) and 6-methylheptacosan-1-ol (**54**), and increased platelet-type crystals on leaf cuticles. This hypothesis is supported by the observation of compact layers of platelet-shaped crystals that have been shown to contribute to various means of biotic and abiotic resistance (e.g., insect deterrence, drought stress management, temperature regulation) [10,12,46,53].

It is of importance to note that, due to difficulty in growing spring wheat cultivars in Colorado fields, Conan and Reeder were grown in the greenhouse and this may have had an influence on wax variation when compared to winter cultivars (Denali and Hatcher) grown in the field. Increased wax content in Conan might be due to a genotype by environment (G × E) interaction, however, no clear trend was observed for either wax content (Figure S1) or crystal composition for plants grown in the field or greenhouse.

### 3.5. A GC-MS Metabolomics Workflow Was Applicable to Assess Epicuticular Wax Variation in Wheat

The metabolomics methods applied to this study included uni- and multivariate statistics of quantitative data. Based on these statistics, variation in cuticular wax content between wheat tissues and among cultivars was concluded (Figure 3). Using similar observations described in the literature, we hypothesized that chemical variation in waxes would result in variation of epicuticular wax crystals on the cuticle, and that observing this correlation would help validate the relevance of our metabolomics model. Data presented in this study support the utility of this method.

The choice of a non-polar solvent for wax extraction shows relevance as hexane yields plant waxes of agronomic interest including alkanes, primary alcohols, fatty acids, ketones, and aldehydes [10,21,22,23]. In the present work, the use of hexane enabled the detection of 263 putative compounds including 58 cuticular waxes. Twenty alkanes, ten primary alcohols, 22 fatty acids, and six ketones were identified. When comparing acids, alcohols and ketones to alkanes of similar chain length, the former display a relatively higher polarity than the latter. The use of hexane thus allowed for the discrimination of metabolites that belong to a broad range of polarities. Moreover, the relatively short exposure of tissues to hexane (i.e., 30 s) necessary to extract a substantial proportion of waxes makes it an important asset for fast, high-throughput protocols with large sample sizes. Still, careful attention must be given to the duration of tissue exposure due to a potential local disruption of the cuticle membrane and release of internal compounds that are not surface waxes [58]. It is likely that the additional 11 non-wax compounds annotated are intracuticular metabolites that result from either the cut-open section of tissue that was immersed, or a slightly excessive exposure to the solvent. Further, even with a large sample set, little variation was observed among replicates of tissues within cultivars (Figure 3), supporting the reproducibility of our method.

The use of GC-MS instrumentation for low molecular weight, volatile compounds such as cuticular waxes is recommended [11,13,24,28,29,30]. Gas chromatography apparatuses include a hot evaporation chamber where samples are injected prior to column separation. A split/splitless sample injection mode is selected based on parameters that include peak resolution, column capacity and set up complexity and aims at reducing (split mode) or not (splitless mode) the sample quantity before transport to the column. While the analytes may suffer from mass discrimination in split mode (high molecular weight compounds do not have enough time to vaporize, and their abundances are consequently less representative), its ease to use and automate, protection from non-volatile compound contamination, less propensity to thermal degradation, and sharper analyte peaks makes it a program of choice for plant wax detection. While splitless mode can result in large peaks with plateaus and tails, split mode generates metabolite profiles that are better suited for quantitative comparisons. Moreover, the GC oven temperature program is critical for separation of wax compounds [24,25,26]. The relatively short temperature ramp used here allowed for quick discrimination of a complex mixture of waxes from a wide range of mass-to-charge ratio values (i.e., 50–650 *m*/*z*) (Table 1 and Table 2), and provided quick sample-to-sample run times, thus reducing analytical error and improving quantitative comparisons among samples. However, it is possible that these technical choices prevented the identification of aldehyde, ester or secondary alcohol waxes, given their chemical properties (e.g., polarity, boiling point, molecular weight).

Further, the R packages XCMS and RAMClust were used to create an accurate matrix of molecular features, each representing a putative metabolite [59,60,61]. XCMS data preprocessing includes several common procedures aimed at curating raw data from metabolite profiling experiments, yet few wax studies incorporate this critical tool. Mass spectrometry instruments deliver complex datasets that need thorough preprocessing. XCMS includes consistent filtering, detection and alignment of *m*/*z* peaks, retention time correction, and peak filling that removes zero values from the dataset, as explained by Smith et al. [61]. Further curation of MS datasets is possible using the R package RAMClust, a deconvolution algorithm [59]. Where XCMS develops a data set by which molecular features are independent, RAMClust groups features into spectral clusters to represent a single metabolite. The RAMClust method can also improve statistical robustness by reducing the number of data points to analyze (i.e., reducing false discoveries), and by establishing new quantitative values for each metabolite by integrating abundance values from all molecular features within a spectral cluster [59]. However, a potential pitfall that rises from spectral matching is the use of non-exhaustive databases. Further collective efforts must be made to create more reliable and complete directories for GC compounds, including the integration of fragmentation patterns for the most common electron impact configurations.

Studies on wax variation among tissues and cultivars of various plant systems incorporate the use of standardized methods of detection and analysis of metabolites. In wheat, common procedures include the use of polar solvents, splitless injection of samples in the GC column, and importantly, rarely incorporate advanced preprocessing data algorithms (e.g., [10,28,62,63]). Our GC-MS metabolomics methods are well developed and have been applied to a broad range of scientific questions and biological systems [22,23,33,34,59]. The methods used in this study utilized GC-MS detection of waxes and metabolomics pre- and post-processing tools that enabled the detection and relative quantitation of 263 putative metabolites. Future work can apply this workflow to evaluate wax variation important to breeding, genetic mapping, and stress resistance in wheat and other major crops.

### 3.6. Conclusions

Our non-targeted GC-MS metabolomics data demonstrate that variation in cuticular wax composition and crystal microstructure exists among tissues and cultivars of common wheat. Leaf surfaces were characterized by high levels of alcohols and stem surfaces showed higher content in β-diketones. While most of the detected compounds were equally distributed among cultivars, two Conan wax alcohols were higher in content than the other cultivars. Further, SEM imaging provided insights in wheat wax microstructural topography and allowed for the identification of two types of epicuticular wax crystals in wheat: platelets and tubules.

## 4. Materials and Methods

### 4.1. Plant Material

Hard red winter wheat (*Triticum aestivum* L. (Poaceae)) cultivars “Hatcher” [64] and “Denali” [65], and hard red spring wheat cultivars “Conan” (PI 607549) and “Reeder” (PI 613586) were used for comparisons of cuticular wax metabolites and epicuticular wax crystals. For metabolomics analyses, winter wheat cultivars were vernalized for eight weeks at 3 ± 2 °C. Spring wheat cultivars were vernalized for 10 days at 3 ± 2 °C to facilitate more synchronous development of the winter and spring wheat cultivars. Vernalized seedlings were planted in 5-inch circular pots in the following mix: seven parts Fafard professional metro mix (45–55% Canadian Sphagnum peat moss, vermiculite, bark, dolomite lime, and wetting agent) (Sun Gro Horticulture, Agawam, MA, USA), two parts coarse perlite, one part Fort Collins loam soil supplemented with aged manure and Osmocote slow release fertilizer (Greenhouse Products Pty Ltd., Princess, South Africa) as per the manufacturer’s recommendation. All plants were grown at the Colorado State University greenhouse, Fort Collins (CO, USA), at 18–24 °C with a photoperiod of ca. 15/9 h light/darkness, bottom watered three times per week, and grouped in a randomized complete block design. Photographs of wheat plants were taken at Zadoks growth stage ca. 55 (i.e., heading stage) [66]. For microscopic observations, winter wheat cultivars were grown in the field (New Raymer, CO, USA) under conventional conditions (e.g., precipitation, temperature, photoperiod), and spring wheat cultivars were grown in the greenhouse under the same conditions used for GC-MS analysis.

### 4.2. Leaf Photographs for Glaucousness

Visual determination of variation in glaucousness on leaf adaxial and abaxial, and stem cuticles was done by photographing five individuals from each of the four wheat cultivars at the same age (Zadoks stage ca. 55). All pictures of biological replicates for each cultivar showed similar glaucousness profiles and one replicate per cultivar was picked at random to create Figure 1. All photographs were recorded on the same day using a Canon EOS Rebel T3 camera (Canon Inc., Ōta, Tokyo, Japan) with identical camera setup, background, and light conditions throughout. Pictures were assembled in Figure 1 using Adobe Photoshop v16.0 (Adobe Systems, San Jose, CA, USA) without any technical adjustment such as manipulation of contrast or saturation.

### 4.3. Metabolite Extraction, Detection by GC-MS, Data Processing and Annotation

A total of 80 plants was used for cuticular metabolite extraction, including 21 biological replicates of Hatcher (11 leaf and 10 stem samples), 19 replicates of Denali (10 leaf and nine stem samples), 20 replicates of Conan (10 leaf and 10 stem samples), and 20 replicates of Reeder (10 leaf and 10 stem samples). Equivalent positions were probed in all four cultivars to account for wax variation between the upper and lower parts of the plants. Stems between the third and fourth internodes (starting from the first internode, also termed peduncle), and the fourth leaf of each plant (starting from the flag leaf), were collected. Waxes and non-wax metabolites were extracted as described by Zhang et al. [22] with the following modifications. Briefly, plain lyophilized leaf pieces (8 mm × 8 mm = area of 64 mm^2^ on both sides = 128 mm^2^ total) and plain lyophilized stems (1.5 mm radius × 85 mm height = 128 mm^2^ total) were dipped into glass vials containing 1 mL of gas chromatography grade hexane (Sigma-Aldrich, Inc., St Louis, MO, USA) [23]. To extract a significant proportion of waxes from the cuticle, samples immersed in hexane were agitated for 30 s on a rotator at 100 rpm. Even though samples were gently shaken, this short period was enough for the solvent to locally corrode the cuticle membrane and release intracuticular compounds along with extracuticular waxes [58], notwithstanding the cut-open section of tissue that was immersed in the solvent and potentially released more intracuticular metabolites. Waxes were therefore referred to as “cuticular waxes” in the abstract, results, discussion and materials and methods sections, while the term “epicuticular wax crystals” was employed for microscopy visualization purposes. Solvents were then decanted into new glass vials. Vial containers and tissues were given a further 10-s rinse with the same amount of hexane, and both solutions were combined in a new vial. Hexane-soluble extracts were then evaporated under a continuous gas nitrogen flow. Metabolites were derivatized by adding 60 µL of a pyridine: *N*-Methyl-*N*-(trimethylsilyl)trifluoroacetamide (MSTFA) solution (1:1, *v*:*v*) and incubating for 30 min at 60 °C. Non-targeted metabolite profiling was performed using gas chromatography-mass spectrometry (GC-MS) as previously described [33]. Briefly, metabolites were detected using a Trace GC Ultra coupled to a Thermo DSQ II mass spectrometer (Thermo Scientific, Waltham, MA, USA). Samples were injected in a 1:10 split ratio twice in discrete randomized blocks. Injection of a pooled quality control was performed every 10 sample injections. High Spearman’s rank correlation coefficients ensured the stability of the instrument over time. Separation occurred using a 30 m TG-5MS column with a film thickness of 0.25 μm (Thermo Scientific), and a 1.2 mL per min helium gas flow rate. The program consisted of 80 °C for 30 s, a ramp of 15 °C per min to 330 °C, and an 8-min hold. Other specifications included inlet temperature held at 280 °C and auxiliary line at 300 °C. Masses between 50–650 *m*/*z* (i.e., mass-to-charge ratio) were scanned at five scans per s after electron impact ionization.

Data files from GC-MS experiment were converted to .cdf format and processed by XCMS in R v3.2.4 (R foundation for Statistical Computing, Vienna, Austria) [61] to create a matrix of molecular features as defined by retention index (RI, Kovats alkane-based index) and mass (*m*/*z*). Upon collection of fatty acid (ranging from C_18_ to C_36_) retention times using AMDIS v2.71 (NIST, Gaithersburg, MD, USA), the Golm Metabolome Database [67,68] was used to obtain the corresponding RI. Retention indices from other chemical classes were deduced using the Kovats RI for temperature ramped columns in AMDIS. Data was deconvoluted into spectral clusters using the R package RAMClust [59]. Critical RAMClust parameters included minimum module size of 2 (if a feature is clustered in a group with less than two features, it will not be exported as a putative compound); “average” linkage (method used to perform fastcluster-based hierarchical clustering), h_max_ = 0.9, st = 4, sr = 5; and features were normalized to total ion current (TIC). The relative quantity of each molecular feature was determined by the mean area of the chromatographic peak among two replicate injections, and spectral clusters were quantified as a weighted abundance of all molecular features in the cluster. Due to TIC normalization and based on the assumption that all extracts have equal metabolite quantities, metabolite abundances were discussed throughout as “content”. Identification of metabolites was performed by matching mass spectra and retention indices to in-house and external databases including NIST 14 [69] and the Golm Metabolome Database (gmd20111121_var5_alk). Confidence levels of annotations were designated based on classification of metabolite annotation by Sumner et al. [70]. In sum, a confidence level of 1 is achieved when two or more forms of data from a given compound match an authentic reference standard, level 2 means a given compound is putatively identified when spectral data or spectra from a database is available but no comparison to a reference standard, and level 3 is assigned when only the compound class can be identified. All annotated metabolites in this study were assigned a level 2 confidence. The .txt document derived from the original .msp file contains metabolite annotation information and is provided in the supplementary materials (Table S1).

### 4.4. Scanning Electron Microscopy

Wheat cultivars Hatcher and Denali were grown in the field (New Raymer, CO, USA), and Conan and Reeder were grown at the Colorado State University greenhouse due to inconsistent growth of spring wheats in Colorado fields. Leaves and stems from each cultivar were collected at heading stage (ca. Zadoks 55). Tissue samples of 15 cm in length were cut (as in the metabolite extraction protocol, stems between the third and fourth internodes and the fourth leaf of each plant were collected) and placed into 10 mL tubes. Small pieces of tissue (8 mm × 8 mm of leaves, and 8 mm × 8 mm of unrolled stems) were then mounted on stubs and placed in an air-dryer for desiccation to prevent sample shrinkage after exposure to the microscope vacuum chamber. Dried samples were coated with a layer of gold (20 nm) using a Hummer VI sputtering system (Anatech Ltd., Springfield, VA, USA). Cuticles were visualized using a JEOL JSM-6500F scanning electron microscope (JEOL, Peabody, MA, USA) set at a beam accelerating voltage of 15 kV. For each cultivar, three tissue types (adaxial leaf surface, abaxial leaf surface, stem) were observed in two plants (*n* = 2 biological replicates). For each tissue type, two structures were observed and each picture was recorded from five different sample areas, for a total of 240 observations.

### 4.5. Statistical Analysis

Metabolite contents were compared using one-way ANOVA for both tissues and cultivars, with a *p* threshold of 0.05. Differences in content among cultivars were further compared using Tukey HSD pairwise comparisons. Benjamini–Hochberg correction was systematically applied across all *t*-tests and ANOVA metabolomics results to account for falsely rejected statistical hypotheses when conducting multiple comparisons, termed “false discovery rate” (FDR) [71]. Principal component analysis (PCA) was conducted on the GC-MS data after mean-centering and UV-scaling using SIMCA v14.1 (MKS Data Analytics, Umea, Sweden). Heat maps were prepared in the R environment v3.2.4 using the heatmap.2 function in the R package gplots, and hierarchical clustering was performed using the hclust function in R. Heat map z scores were calculated using the mean and standard deviation of metabolite content: z = (X − μ)/σ, where X is the relative content of a metabolite, μ is the mean content for the metabolite across all samples and σ is the standard deviation among all samples.

## Figures and Tables

**Table 1:** Chemical composition of cuticular waxes detected on wheat leaves and stems.

#	IUPAC Name (Synonym)	Retention Index	ANOVA *p*-Value ^†^		Association	Reference
			Tissue	Cultivar		
Alkanes						
**1**	Eicosane	2000	^‡^	0.6	Plant–insect int.	[10]
**2**	Tricosane	2300	0.13	^‡^	Chemotaxonomy	[24]
**3**	4-Methyldocosane	2334	0.33	^‡^		
**4**	6-Methyldocosane	2336	^‡^	^‡^		
**5**	Pentacosane	2500	^‡^	0.77	Chemotaxonomy	[24]
**6**	Hexacosane	2600	^‡^	0.17	Chemotaxonomy	[24]
**7**	5-Methylpentacosane	2641	^‡^	0.63		
**8**	7-Methylpentacosane	2645	^‡^	0.63		
**9**	Heptacosane	2700	^‡^	0.56	Chemotaxonomy	[24]
**10**	4-Methylhexacosane	2727	^‡^	0.4		
**11**	12-Methyloctacosane	2912	^‡^	^‡^		
**12**	14-Methyloctacosane	2915	^‡^	^‡^		
**13**	Triacontane	3000	^‡^	^‡^	Chemotaxonomy	[24]
**14**	Hentriacontane	3100	0.12	0.05	Plant–insect int.	[10]
**15**	7-Methylhentriacontane	3111	^‡^	^‡^		
**16**	13-Methylhentriacontane	3124	^‡^	^‡^		
**17**	Tetratriacontane	3400	0.27	^‡^	Chemotaxonomy	[24]
**18**	Pentatriacontane	3500	0.61	0.16	Chemotaxonomy	[24]
**19**	Tetracontane	4000	^‡^	^‡^	Plant–insect int.	[36]
**20**	Dotetracontane	4200	^‡^	^‡^		
Fatty acids						
**21**	Heptanoic acid (enanthic acid)	1044	0.25	0.3		
**22**	Tetradecanoic acid (myristic acid)	1499	^‡^	0.68	Antibiotic	[37]
**23**	Hexadecenoic acid	1602	0.51	0.57		
**24**	Hexadecanoic acid (palmitic acid)	1623	^‡^	0.9	Plant–insect int.	[10]
**25**	(9*Z*,12*Z*,15*Z*)-Octadeca-9,12,15-trienoic acid (α-linolenic acid)	1755	^‡^	0.13	Oil production	[38]
**26**	Octadecanoic acid (stearic acid)	1765	0.25	0.77	Plant–insect int.	[39]
**27**	10-Methylheptadecanoic acid	1768	0.12	0.61		
**28**	Nonadecanoic acid	1825	0.67	0.3	Plant–insect int.	[40]
**29**	Eicosanoic acid (arachidic acid)	1911	^‡^	0.52		
**30**	Docosanoic acid (behenic acid)	2103	^‡^	0.05	Oil production	[41]
**31**	Tricosanoic acid	2166	^‡^	0.77		
**32**	Tetracosanoic acid (lignoceric acid)	2246	^‡^	0.09	Major wax precursor	[42]
**33**	Hexacosanoic acid (cerotic acid)	2404	^‡^	0.06	Allelopathy	[43]
**34**	Heptacosanoic acid	2497	0.07	0.17		
**35**	Octacosanoic acid (montanic acid)	2563	^‡^	0.46		
**36**	25-Methylheptacosanoic acid	2569	0.07	0.17		
**37**	Nonacosanoic acid	2647	0.61	0.25		
**38**	23-Triacontenoic acid	2728	^‡^	0.54		
**39**	12-Methylnonacosanoic acid	2731	^‡^	0.77		
**40**	Triacontanoic acid (melissic acid)	2741	^‡^	0.32		
**41**	Hentriacontanoic acid	2796	^‡^	0.32		
**42**	Tetratriacontanoic acid (geddic acid)	3037	^‡^	^‡^	Plant–insect int.	[18]
Ketones						
**43**	Nonane-4,6-dione	1124	^‡^	0.33		
**44**	Nonacosane-12,14-dione	2689	^‡^	0.41	Major leaf wax	[44]
**45**	Triacontane-12,14-dione	2698	^‡^	0.21		
**46**	Hentriacontane-14,16-dione	2735	^‡^	0.39	Glaucousness	[45]
**47**	Tritriacontan-3-one	2839	0.93	^‡^		
**48**	Pentatriacontan-2-one	2881	^‡^	0.54		
Primary alcohols						
**49**	Docosan-1-ol (behenyl alcohol)	2044	^‡^	^‡^		
**50**	Tricosan-1-ol	2112	0.79	^‡^		
**51**	Hexacosan-1-ol	2325	^‡^	^‡^	Plant–insect int.	[10]
**52**	Heptacosan-1-ol	2404	0.9	^‡^		
**53**	Octacosan-1-ol	2467	^‡^	0.77	Plant–insect int.	[10]
**54**	6-Methylheptacosan-1-ol	2498	^‡^	^‡^		
**55**	Triacontan-1-ol (melissyl alcohol)	2668	^‡^	0.63		
**56**	Hentriacontan-1-ol	2703	^‡^	0.25		
**57**	22-Methylhentriacontan-1-ol	2780	^‡^	0.41		
**58**	Tritriacontan-1-ol	2855	^‡^	^‡^		

^†^ Each *p*-value was calculated using one-way ANOVA (factors of cultivar and tissue) and adjusted by a Benjamini–Hochberg correction. The Association column refers to biological function with which metabolites are associated. Abbreviations/Notations: # = compound ID; **^‡^** = *p* < 0.05; int. = interaction.

**Table 2:** Chemical composition of non-wax cuticular metabolites from wheat leaves and stems.

#	Class	IUPAC Name (Synonym)	RI	ANOVA *p*-Value ^†^	
				Tissue	Cultivar
**59**	Carboxylic acid	2-Hydroxybutanedioic acid (malic acid)	1192	0.19	^‡^
**60**		Tetradecanedioic acid	1471	0.14	0.34
**61**		5-(Dioctadecylamino)-5-oxo-pentanoic acid	2589	^‡^	0.3
**62**	Carbohydrate	(3 *S*,4*S*,5*S*,6*R*)-6-(Hydroxymethyl)oxane-2,3,4,5-tetrol (d-mannose)	1602	0.23	^‡^
**63**		(2 *R*,3*R*,4*S*,5*S*,6*R*)-2-[(2*S*,3*S*,4*S*,5*R*)-3,4-Dihydroxy-2,5-bis(hydroxymethyl)Oxolan-2-yl]oxy-6-(hydroxymethyl)oxane-3,4,5-triol (sucrose)	2140	0.89	0.3
**64**	Primary amine	Hexadecan-1-amine	854	^‡^	0.61
**65**	Polycyclic ketone	1,2-Diphenyl-2-buten-1-one	2184	0.15	0.57
**66**		3-Acetyl-8-methoxy-2-methyl-1H-naphtho[2,1-b]pyran-1-one	2389	^‡^	0.14
**67**	FAME	Methyl-6-(1-hydroxyethyl)phenazine-1-carboxylate(saphenic acid methyl ester)	2885	^‡^	0.24
**68**	Flavonoid	3,5-Dihydroxy-4′′,7-dimethoxyflavone (pilloin)	2287	^‡^	0.92
**69**	Sterol	(3 *S*,8*S*,9*S*,10*R*,13*R*,14*S*,17*R*)-17-[(2*R*,5*R*)-5-Ethyl-6-methylheptan-2-yl]-10,13-dimethyl-2,3,4,7,8,9,11,12,14,15,16,17-dodecahydro-1H-cyclopenta[a]phenanthren-3-ol (β-sitosterol)	2801	^‡^	0.4

^†^ Each *p*-value was calculated using one-way ANOVA (factors of cultivar and tissue) and adjusted by a Benjamini–Hochberg correction. Abbreviations/Notations: # = compound ID; RI = retention index; **^‡^** = *p* < 0.05; FAME = fatty acid methyl ester.

## Supplementary Materials

The following are available online at http://www.mdpi.com/1422-0067/19/2/249/s1. Figure S1. Wax density.docx provides a semi-quantitative analysis of wheat epicuticular wax density using image processing tools on SEM micrographs; Table S1. Wax metabolite annotations.txt provides detailed information on detected metabolites.

## Abbreviations

ANOVA	Analysis of variance
FDR	False discovery rate
GC-MS	Gas chromatography-mass spectrometry
HSD	Honest significant difference
PC	Principal component
PCA	Principal component analysis
RI	Retention index
SEM	Scanning electron microscope
TIC	Total ion current
