# Supplementary material for: GC-MS Metabolomics to Evaluate the Composition of Plant Cuticular Waxes for Four Triticum aestivum Cultivars
Source: Int J Mol Sci. 2018 Jan 23;19(2):249. doi: 10.3390/ijms19020249 (PMC5855543; doi:10.3390/ijms19020249)
Supplement: Supplementary file 1 [file ijms-19-00249-s001.zip › ijms-253287-final-supplementary materials/Figure S1 - Wax density.docx]

*Association of Epicuticular Wax Content and Crystal Microstructure in Wheat*

The qualitative variation observed using SEM was validated by quantifying epicuticular wax content (percent wax abundance on the cuticle total surface) using image processing software. The software ImageJ v1.51d was used to quantify wax surface content on low magnification pictures (×140). Briefly, after conversion of images to red-green-blue (RGB) stack, luminosity was manually adjusted to select waxes only (white/greyish pixels). Wax content was then measured and expressed as percentage of wax coverage across the entire tissue considered. The analysis revealed variation in content among the three tissue types (ANOVA, Tukey HSD post-hoc *p* = 2 × 10^-16^) (Figure S1). When pooling the four cultivars, the mean wax coverages were 44.4 ± 1.2% for the adaxial leaf surface, 28.8 ± 1.8% for the abaxial leaf surface, and 14.4 ± 0.7% of the whole stem. Reeder adaxial leaf surface demonstrated the highest amount of epicuticular wax crystals (55.6 ± 2.2% coverage of leaf surface), compared to Reeder abaxial leaf surface (37.6 ± 1.5%) and stems (12.9 ± 1.7%). This distribution of wax crystals (adaxial >abaxial >stem) was consistent for each cultivar except Conan, whose abaxial leaf surface had increased crystal microstructures attributed to platelets.


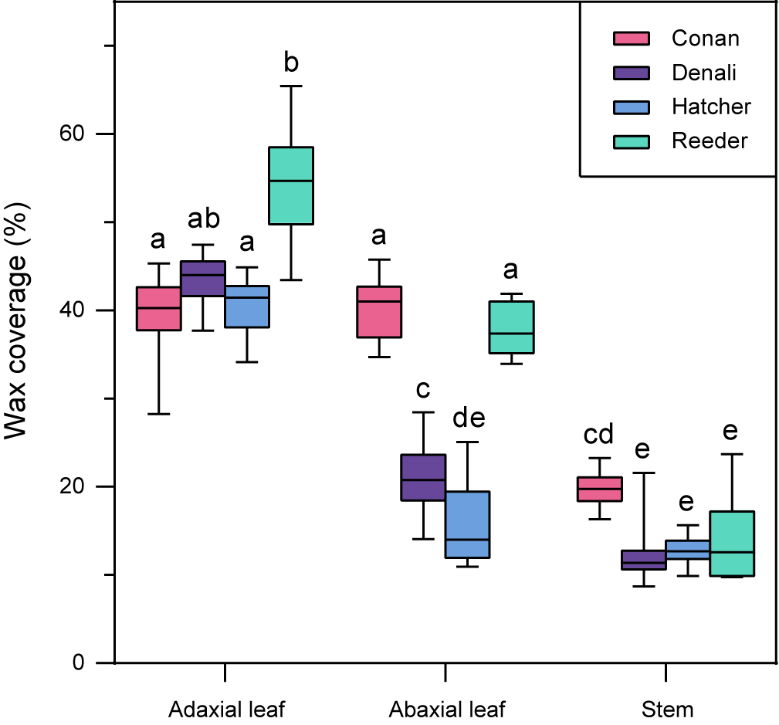


**Figure S1.** Epicuticular wax content among cultivars and tissue types. Wax contents are expressed as the percentage of the total surface covered, and reported as the mean content for each cultivar within each tissue (*n* = 2 biological replicates and *n* = 5 technical replicates per cultivar per tissue). Lower case letters indicate Tukey HSD post-hoc groupings (ANOVA, Tukey HSD post-hoc *p* <0.05).
